# Paramylon from *Euglena gracilis* Prevents Lipopolysaccharide-Induced Acute Liver Injury

**DOI:** 10.3389/fimmu.2021.797096

**Published:** 2022-01-11

**Authors:** Yunhao Xie, Jin Li, Huan Qin, Qing Wang, Zixi Chen, Chengyu Liu, Ling Zheng, Jiangxin Wang

**Affiliations:** ^1^ Hubei Key Laboratory of Cell Homeostasis, College of Life Sciences, Wuhan University, Wuhan, China; ^2^ Shenzhen Key Laboratory of Marine Bioresource and Eco-Environmental Science, Shenzhen Engineering Laboratory for Marine Algal Biotechnology, Guangdong Provincial Key Laboratory for Plant Epigenetics, College of Life Sciences and Oceanography, Shenzhen University, Shenzhen, China; ^3^ College of Food Engineering and Biotechnology, Hanshan Normal University, Chaozhou, China; ^4^ Department of Transfusion Medicine, Wuhan Hospital of Traditional Chinese and Western Medicine, Tongji Medical College, Huazhong University of Science and Technology, Wuhan, China

**Keywords:** acute liver injury, *Euglena gracilis*, paramylon, sonicated and alkalized paramylon, inflammation

## Abstract

Acute liver injury (ALI) is a life-threatening syndrome with high mortality and lacks effective therapies. Rodents under LPS (lipopolysaccharide)/D-Gal (D-galactosamine) stress mimic ALI by presenting dramatically increased inflammation and cell death in the liver. *Euglena gracilis*, functioning like dietary fiber, is commonly used as a paramylon (Pa)-rich nutritional supplement that has various biological effects such as regulating immune system, anti-obesity, and anti-tumor. Here, we found that Pa or sonicated and alkalized paramylon (SA-Pa) alleviated the LPS/D-Gal-induced hepatic histopathological abnormalities in mice. Compared with Pa, SA-Pa had lower molecular weights/sizes and showed better efficacy in alleviating injury-induced hepatic functions, as well as the transcriptional levels of inflammatory cytokines. Moreover, SA-Pa treatment promoted M2 macrophage activation that enhanced the anti-inflammatory function in the liver, and downregulated STAT3 target genes, such as *Fos, Jun*, and *Socs3* upon the injury. Meanwhile, SA-Pa treatment also alleviated apoptosis and necroptosis caused by the injury. Our results demonstrated that SA-Pa efficiently protected the liver from LPS/D-Gal-induced ALI by alleviating inflammation and cell death.

## Introduction

Acute liver injury (ALI), a life-threatening syndrome with a near 80% mortality rate, is mainly characterized by rapidly destroyed hepatic function accompanied with multiple organ failure (1, 2). Without timely treatment, ALI promptly progresses and liver transplantation is ultimately required (3). Presently, few preventive or therapeutic strategies are available for ALI (4); thus, novel effective therapeutic strategies are desired.

LPS (lipopolysaccharide), which stimulates inflammatory responses, is an endotoxin existing in the outer membrane of Gram-negative bacteria (5). LPS binds to the Toll-like receptor (TLR) on the Kupffer cells to induce the transcription of inflammatory factors, such as tumor necrosis factor α (TNFα), interleukin-1β (IL-1β), and interleukin-6 (IL-6), which subsequently induce hepatocyte apoptosis (6). D-galactosamine (D-Gal) enhances the hepatotoxicity of LPS *via* inhibiting RNA and protein synthesis (7). Therefore, LPS/D-Gal is commonly used to induce ALI in animal models with severe hepatic inflammation similar to human hepatitis (8, 7).

*Euglena gracilis* produces and stores a unique polysaccharide named paramylon (Pa). Under optimal heterotrophic culture conditions, the Pa content can reach up to 50%–70% of the dried biomass of *E. gracilis* (9). Pa is a water-insoluble linear (unbranched) β-(1,3)-glucan polysaccharide polymer with a molecular mass of about 500 kDa (10). Due to its high molecular mass, Pa has low solubility and poor bioavailability (11, 12) however, alkalization treatment reduces its molecular mass to 12 kDa, which greatly increases solubility and bioavailability *in vitro* (13).

Previous studies have reported a variety of biological functions of *Euglena* or Pa, including effects on the immune system (14–17). Moreover, functioning like dietary fiber, Pa has been shown to mitigate obesity by showing reduced serum LDL-cholesterol level and abdominal fat accumulation, as well as improved postprandial glucose level (18). Moreover, Pa has been shown to have anti-tumor activity in mouse against preneoplastic colonic aberrant crypt foci (19). In a rodent model of type 2 diabetes, Pa has been demonstrated to alleviate hepatic fibrosis (20). However, the effects of Pa and its alkalization form (SA-Pa) on inflammation and cell death, which are closely associated with ALI, remain unclear.

Here, we found that Pa or SA-Pa treatment alleviated LPS/D-Gal-induced hepatic damages, with SA-Pa showing higher efficacy. Moreover, SA-Pa treatment promoted M2 macrophage activation and mitigated apoptosis and necroptosis upon injury. Therefore, SA-Pa may be further explored as a nutritional supplement against ALI.

## Materials and Methods

### Reagents

LPS and D-Gal were purchased from Sigma-Aldrich (St. Louis, MO). TUNEL assay kit was from Beyotime Biotechnology (Shanghai, China). Antibodies against p-STAT3 (#9134), STAT3 (#4904), F4/80 (#D2S9R), p-RIP3 (#57220), Caspase-3, and C-Caspase-3 (#9665) were obtained from Cell Signaling Technology (Danvers, MA); antibodies against Ly6G (#551459) and HSP90 (#51-9001986) were obtained from BD Pharmingen (Franklin Lakes, NJ); antibodies against CD3 (#GAO45229), Caspase-8 and C-Caspase-8 (#13423), and CD163 (#33560) were obtained from Gene Tech (San Francisco, CA), Proteintech (Rosemont, IL), and Santa Cruz (Dallas, TX), respectively.

### Preparation and Extraction of Paramylon

*E. gracilis* CCAP 1224/5Z was purchased from CCAP (Culture Collection of Algae and Protozoa) and maintained in Wang’s lab in Shenzhen University. *E. gracilis* were grown in glucose-rich EM medium [1.8 g/L NH_4_Cl, 0.6 g/L KH_2_PO_4_, 0.6 g/L MgSO_4_, 60 mg/L Urea, 0.02 g/L CaCl_2_, 0.48 mg/L Na_2_EDTA, 2 mg/L Fe_2_(SO_4_)_3_, 25 g/L glucose, 0.01 mg/L vitamin B_1_ (VB_1_), 0.0005 mg/L VB_12_, 20 mg/L CuSO_4_·5H_2_O, 0.4 g/L ZnSO_4_·7H_2_O, 1.3 g/L Co (NH_3_)·H_2_O, and 1.6 g/L MnCl_2_·4H_2_O] under darkness at 27°C. Five days later, *E. gracilis* was collected and washed twice with deionized water. Ninety-five percent alcohol was used to break up cells and extract pigments, and alcohol extract was centrifuged at 5,500 *g* for 5 min and the precipitate was collected. To remove lipids and proteins, the precipitate was solubilized in 1% (w/v) sodium dodecyl sulfate at 85°C for 1 h. Pa was then precipitated by centrifugation at 5,000 *g* for 5 min.

To obtain sonicated and alkalized paramylon (SA-Pa), the pellet was further dissolved in 0.5 M NaOH, two volumes of cold 98% ethanol were added, and the mixture was centrifuged at 12,000 *g* for 10 min at 4°C. Ethanol precipitation was repeated once and the final pellet was suspended in deionized water with pH adjusted to 7.0. The suspension was ultrasonicated on ice for 12 min (12 cycles, each with 48 s ultrasonication and 12 s interval) to obtain SA-Pa. Pa and SA-Pa were stored at −20°C before use.

### Animals, Treatments, and Biochemical Measurements

Male C57BL/6 mice were obtained from Hubei Center for Disease Control and Prevention. Mice were randomly assigned into 4 groups, namely, the control, LPS/D-Gal, Pa treatment, and SA-Pa treatment group. In the Pa or SA-Pa treatment group, mice were pretreated with 400 mg/kg body weight (BW) Pa or SA-Pa (both dissolved in water) by gavage at 2 h before LPS/D-Gal injury, while the LPS/D-Gal group was pretreated with equal volume of water. LPS (3 mg/kg BW)/D-Gal (200 mg/kg BW) was intraperitoneally injected into mice to induce ALI. Blood was collected at 6, 9, 12, and 24 h after injection, and serum was harvested by centrifuging the blood at 2,000 *g* for 15 min. Mice were sacrificed at 24 h after the injection. Animals were handled according to the Guidelines of the China Animal Welfare Legislation as approved by the Committee on Ethics in the Care and Use of Laboratory Animals, College of Life Sciences, Wuhan University (approval number: WDSKY0201705-2). Serum alanine aminotransferase (ALT) and aspartate aminotransferase (AST) levels were measured by the respective kit purchased from Jiancheng Bioengineering (Nanjing, China) following the manufacturer’s instruction.

### Histological Analysis, Immunochemistry Staining, and TUNEL Assay

Fresh mouse liver was collected, embedded in paraffin, and sectioned as we previously described (21). One of the liver sections for each sample was stained with hematoxylin and eosin (H&E) for histopathological examination. For immunohistochemical studies, sections were antigen retrieved in citrate buffer (0.01 M sodium citrate, pH 6.0) and incubated with 3% H_2_O_2_ for 5 min to quench endogenous peroxidase activity. After blocking with 2% bovine serum albumin (Amresco, Solon, OH) (22), primary antibodies for F4/80, CD163, Ly6G, or CD3 were applied at room temperature overnight. After washing, sections were incubated with biotinylated secondary antibody (Vector Laboratories, Burlingame, CA). Positive staining was visualized using DAB substrate (Cwbiotech, Beijing, China) followed by the ABC kit (Vector Laboratories). Positively stained areas or cells were quantified using Image-Pro Plus software (Media Cybernetics, Rockville, MD) based on 4–6 different randomly taken fields per sample. For TUNEL assay, a one-step TUNEL apoptosis assay kit (Beyotime Biotechnology, China) was used and performed following the manufacturer’s instruction. TUNEL-positive cells were manually counted and quantitated as we previously reported (21).

### Western Blots

Fresh livers were grounded in ice-cold RIPA buffer (Beyotime Biotechnology) and protein concentrations were determined using the BCA Protein Assay Kit (Cwbiotech). A total of 20–60 mg of protein per sample was separated by SDS-PAGE and electroblotted onto PVDF membrane (Merck Millipore, Darmstadt, Germany) for immunodetection. Primary antibodies were applied at 4°C overnight. After washing, PVDF membranes were incubated with horseradish peroxidase-conjugated secondary antibody (Bio-Rad Laboratories, Hercules, CA). Images of protein bands detected by the antibodies were obtained by the BeyoECL Plus Kit (Beyotime Biotechnology) following different exposure times in a dark chamber of Imager Kwide Quant (Kindle Biosciences, Greenwich, CT) and analyzed by a Kwide Quant Analyzer. Cleaved caspase 3 and cleaved caspase 8 were normalized to total caspase 3 and total caspase 8, respectively; p-STAT3 was normalized to total STAT3, while other targeted protein levels were quantitated relative to the internal control in the same sample as we previously reported (23).

### Quantitative Real-Time PCR

Total RNA was extracted from the livers using RNAiso Plus (TaKaRa Biotechnology, Japan). Total RNA (3 µg) was reverse transcribed into cDNA using the M-MLV first-strand synthesis system (Invitrogen Life Technologies, Carlsbad, CA). The primer sequences were listed as the following: *Il-6*, forward CACTTCACAAGTCGGAGGCT reverse CTGCAAGTGCATCATCGTTGT; for *Il-8*, forward GCACTTGGGAAGTTAACGCA reverse GCACAGTGTCCCTATAGCCC; for *Il-1β*, forward GCAACTGTTCCTGAACTCAACT reverse ATCTTTTGGGGTCCGTCAACT; for *Tnfα*, forward GACGTGGAACTGGCAGAAGAG reverse ACCGCCTGGAGTTCTGGAA; for *Fos*, forward TACTACCATTCCCCAGCCGA reverse GCTGTCACCGTGGGGATAAA; for *Jun*, forward GCACATCACCACTACACCGA reverse GGGAAGCGTGTTCTGGCTAT; for *Socs3*, forward GCCTTTCAGTGCAGAGTAGTG reverse AAGAGCAGGCGAGTGTAGAG; for *Il-10*, forward GCTATGCTGCCTGCTCTTACT reverse CCTGCTGATCCTCATGCCA. qPCR was performed using the Monad Selected q225 Real-Time PCR System (Monad Biotech, Wuhan, China) with *Rn18* as the internal control as we previously described (24), with the relative difference of targeted gene expressed as fold change calculated by the 2^−ΔΔCT^ method.

### Statistical Analysis

GraphPad Prism (version 7.0) was used for statistical calculations. Data were expressed as mean ± SD. All data were analyzed using the non-parametric Kruskal–Wallis test followed by the Mann-Whitney test for comparisons of more than 2 groups, while the Mann–Whitney test was used for comparisons of 2 groups. Differences were considered statistically significant when *p* < 0.05.

## Results

### Characterization of Pa and SA-Pa

**Figure 1:**
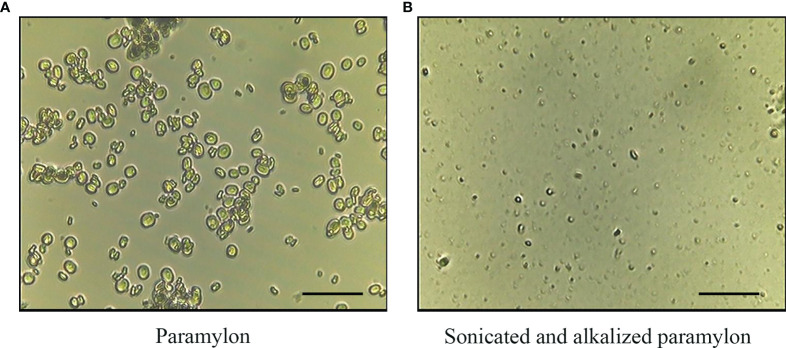
Light microscopy observation of Pa and SA-Pa. Light microscopy (×400) observation of **(A)** untreated paramylon (Pa), and **(B)** ultrasonically treated alkalizated paramylon (SA-Pa) (scale bar = 5 µm).

Morphological difference between Pa and SA-Pa particles was observed under a microscope. Majority of purified paramylon particles have sizes of ~5 µm with some aggregations. Obvious size reduction was observed in SA-Pa particles as 1-2 µm (**Figure 1**).

### Pa and SA-Pa Alleviates LPS/D-Gal-Induced ALI

**Figure 2:**
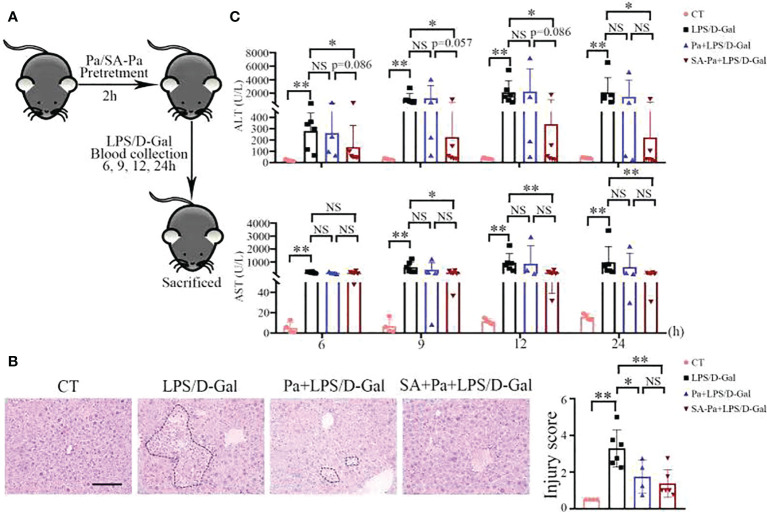
SA-Pa or Pa protected the liver from the LPS/D-Gal induced injury. **(A)** Experimental design for LPS/D-Gal injury with or without Pa/SA-Pa pretreatment. **(B)** Respective H&E staining and quantitative results (scale bar = 100μm). **(C)** Serum levels of ALT and AST. (CT group, n = 4; LPS/D-Gal group, n = 6; Pa group, n = 4; SA-Pa group, n = 6; NS p > 0.05; *p < 0.05; **p < 0.01)

To investigate whether Pa and SA-Pa can protect the liver from LPS-induced ALI, mice were pretreated with Pa or SA-Pa 2 h before LPS/D-Gal injury (**Figure 2A**). Pa or SA-Pa pretreatment significantly suppressed the LPS/D-Gal-induced histopathological abnormalities in the liver as demonstrated by H&E staining (**Figure 2B**). Compared with the control group, LPS/D-Gal induced elevated liver injury demonstrated by hepatocyte necrosis (non-nucleus hepatocytes) and immune cell infiltration, which were alleviated by the SA-Pa or Pa treatment (**Figure 2B**).

Serum ALT and AST levels are sensitive indicators of hepatocellular injury (25) and thus were used to evaluate hepatic function at different times after LPS/D-Gal injury. The levels of ALT/AST were dramatically increased from 6 to 24 h after LPS/D-Gal injection, indicating liver damage (**Figure 2C**). Consistent with pathological findings, the ALT/AST levels of the SA-Pa-treated group were significantly lower than those of the LPS/D-Gal group, while there was a trend in lowering ALT level in the SA-Pa-treated group compared with that of the Pa-treated group at early time points after the injury (**Figure 2C**).

### Pa/SA-Pa Downregulates Inflammatory Genes in the LPS/D-Gal Injured Liver

**Figure 3:**
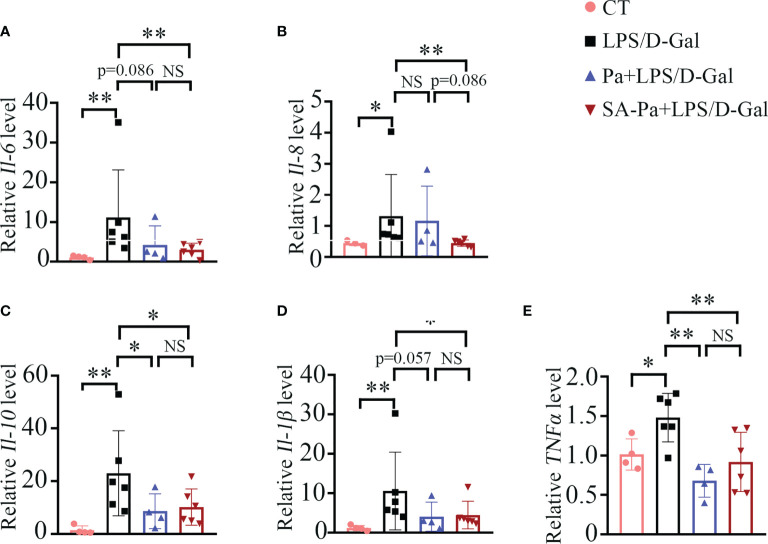
SA-Pa inhibited the transcriptional levels of inflammatory cytokines after the LPS/D-Gal injury. **(A–E)** qPCR results of the indicated genes in the liver of different groups (CT group, n = 3-4; LPS/D-Gal group, n = 6; Pa group, n = 4; SA-Pa group, n = 6; NS p > 0.05; *p < 0.05; **p < 0.01).

In LPS/D-Gal-induced ALI, hepatocyte death leads to the release of damage-associated molecular patterns (DAMPs), which trigger innate immune response and the production of pro-inflammatory mediators such as TNFα, IL-1, and IL-6 (26). IL-8 is one of the key pro-inflammatory cytokines involved in modulating the inflammatory response, and elevated levels of IL-8 are associated with liver injury (25). IL-10 has been reported to be triggered by LPS (27), and elevated serum IL-10 was positively correlated with degree of liver inflammation (28). Thus, we examined the transcript levels of *Il-6*, *Il-8*, *Il-10*, *Il-β* and *TNFα* to analyze the influence of Pa or Sa-PA treatment on the inflammatory responses. Upon LPS/D-Gal injury, evidently increased transcriptions of these inflammatory genes were observed (**Figure 3**). Moreover, SA-Pa treatment significantly attenuated all these injury-induced upregulations, while Pa treatment showed no obvious effect on *Il-6* level, a trend of inhibitory effect on *Il-8* and *Il-β* levels, as well as significant inhibitory effects on *Il-10* and *TNFα* levels (**Figure 3**). Furthermore, compared to Pa treatment, SA-Pa treatment showed a trend in reducing *Il-8* level upon similar injury (**Figure 3B**).

### SA-Pa Activates M2 Macrophages and T Cells in LPS/D-Gal-Induced ALI

**Figure 4:**
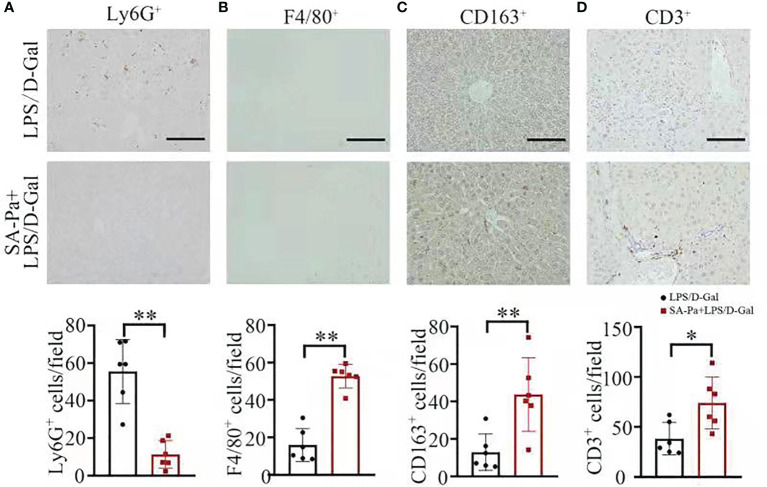
SA-Pa affected the infiltration of different immune cells after the LPS/D-Gal injury. **(A–D)** Representative pictures for Ly6G^+^, F4/80^+^, CD163^+^, and CD3^+^ staining in the liver section (top) and quantitative results (bottom) (LPS/D-Gal group, *n* = 6; SA-Pa group, *n* = 6; *p < 0.05; **p  < 0.01; scale bar = 100 μm).

We next examined the type of infiltrated immune cell in LPS/D-Gal induced-ALI, and to what degree these immune cell infiltrations were affected by Pa or SA-Pa treatment. Immunohistochemical staining of mouse liver tissue suggested that the number of Ly6G-positive cells, a marker of neutrophils (29), was significantly lower in the Pa- or SA-Pa-treated group compared with that of the LPS/D-Gal group (**Figure 4A** and **Supplementary Figure 1A**). Moreover, the number of F4/80-positive macrophages (30) was significantly higher in the SA-Pa- but not Pa-treated group than that of the LPS/D-Gal group (**Figure 4B** and **Supplementary Figure 1B**). Since F4/80-positive cells include both M1 and M2 types of macrophages, which respectively promotes or inhibits inflammation (31), we further evaluated the number of CD163-positive cells, a marker for M2 macrophages (32). The results showed that CD163-positive cells were significantly increased in the liver of the SA-Pa- but not the Pa-treated group (**Figure 4C** and **Supplementary Figure 1C**), suggesting promoted M2 macrophage activation and enhanced anti-inflammatory function in injured liver. However, the number of CD3-positive cells, a marker of T cell (33), was higher in the SA-Pa- but not Pa-treated group than that of the LPS/D-Gal group (**Figure 4D** and **Supplementary Figure 1D**). Therefore, SA-Pa was more effective than Pa in activating M2 macrophages and T cells in LPS/D-Gal-induced ALI.

### SA-Pa Inhibits Transcription Activity of STAT3 in LPS/D-Gal-Induced ALI

**Figure 5:**
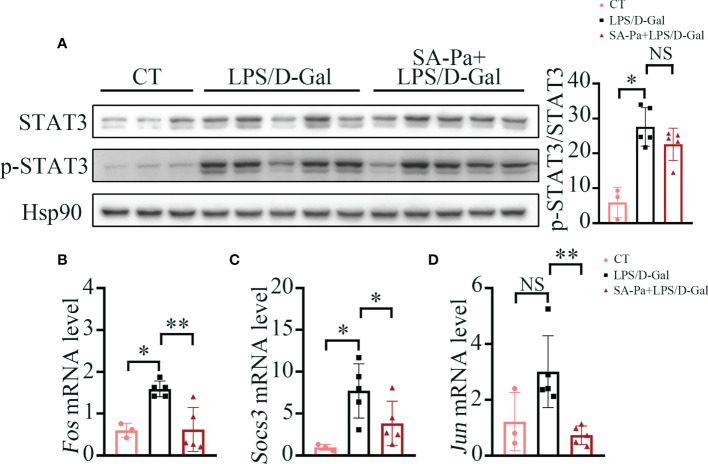
SA-Pa inhibited the transcription levels of STAT3-targeted genes after the LPS/D-Gal injury. **(A)** Western blots and quantitative results for p-STAT3/STAT3 in the liver of indicated groups. (B-D) qPCR results of the indicated genes in the liver of indicated groups (CT group, n = 3; LPS/D-Gal group, n = 5; SA-Pa group, n = 5; NS p > 0.05; *p > 0.05; **p < 0.01).

Under LPS/D-Gal stress, SA-Pa treatment significantly downregulated the mRNA level of *Il-6* in the liver. Since Il-6*-*induced STAT3 activation promotes acute-phase protein production and further contributes to systemic activation of immune responses (34), we next investigated the protein levels of STAT3 and p-STAT3 (activate form of STAT3) that were affected by the treatment. Total STAT3 and p-STAT3 levels were significantly upregulated in the liver after LPS/D-Gal injury; neither SA-Pa nor Pa treatment showed a downregulation of p-STAT3/STAT3 in the liver (**Figure 5A** and **Supplementary Figure 2A**). However, we further found that the transcriptional levels of STAT3 target genes, such as *Fos*, *Socs3*, and *Jun*, were significantly lower in the SA-Pa-treated group, but not the Pa-treated group, than those of the LPS/D-Gal group (**Figures 5B–D** and **Supplementary Figures 2B–D**). It suggested that SA-Pa may mitigate ALI by alleviating STAT3 transcriptional regulation under LPS/D-Gal injury.

### SA-Pa Attenuates LPS/D-Gal-Induced Hepatocyte Apoptosis and Necroptosis

**Figure 6:**
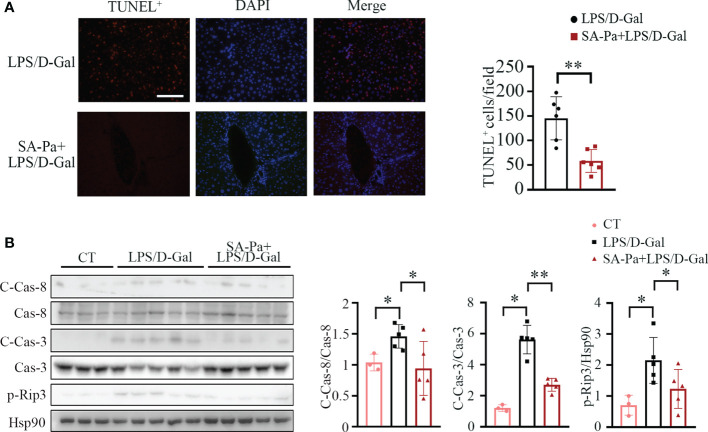
SA-Pa attenuated cell death after the LPS/D-Gal injury. **(A)** Representative pictures for TUNEL^+^ staining in the liver (left) and quantitative results (right). **(B)** Western blots (left) and quantitative results (right) for C-Casp-8/Cas-8 (cleaved-Caspase-8/Caspase-8), C-Cas-3/Cas-3, and p-Rip3 levels. (CT group, *n* = 3; LPS/D-Gal group, n = 5–6; SA-Pa group, n = 5–6; *p < 0.05; **p < 0.01; scale bar = 100 μm).

Liver of LPS/D-Gal-treated mice often show apoptosis and necroptosis (35); we next investigated whether Pa or SA-Pa treatment could alleviate these cell deaths. TUNEL staining, which labels the exposed DNA termini due to apoptosis (36), showed that SA-Pa, but not Pa, treatment significantly reduced the injury-induced incidence of hepatocyte apoptosis (**Figure 6A** and **Supplementary Figure 3A**). Consistently, the level of cleaved caspase-3 (C-Cas-3), the marker of apoptosis, was enhanced in the LPS/D-Gal injured group, but was obviously attenuated in the SA-Pa-treated group, but not the Pa-treated group, under similar injury (**Figure 6B** and **Supplementary Figure S3B**), while either Pa or Sa-Pa treatment significantly reduced the level of cleaved caspse-8 (C-Cas-8) (**Figure 6B** and **Supplementary Figure S3B**). As for necroptosis, SA-Pa but not Pa treatment significantly reduced the p-Rip3 level, a necroptosis marker, upon LPS/D-Gal injury (**Figure 6B** and **Supplementary Figure 3B**).

## Discussion

Morphologically and functionally, LPS/D-Gal-induced liver injury closely resembles human ALI. This model is thus widely used to study ALI pathogenesis and identify novel therapeutic chemicals (37). In the present study, we found that SA-Pa from *E. gracilis* effectively alleviated LPS/D-Gal-induced ALI in mice. SA-Pa treatment only effectively alleviated the injury-induced serum AST and ALT levels; moreover, the present study identified for the first time the anti-apoptosis and anti-necroptosis effects of SA-Pa on LPS/D-Gal-induced liver damage. Regarding the superior protection effect of SA-Pa, a possible explanation is that paramylons with a large molecular weight of over 500 kDa have low solubility and poor bioavailability, while alkalization treatment reduces the molecular weight of SA-Pa (38). Moreover, alkalization and ultrasonic treatment destroy the multi-stranded helix of β-(1,3)-glucan and of Pa to short-chain soluble β-1,3-glucan (39), making SA-Pa more soluble and absorbable (14), which may be behind the better protection effects of SA-Pa treatment compared to Pa treatment.

Liver contains a large number of innate and adaptive immune cells, including macrophages (resident Kupffer cells and circulating monocytes), B cells, T cells, and NK cells, which specialize in detection and capture of pathogens from the blood such as LPS (40). Upon LPS/D-Gal-induced injury, Kupffer cells activate and secrete a large variety of cytokines and chemokines (41). Circulating monocytes are recruited to the injured liver *via* chemokine signals (42). A previous study also indicates that β-glucan from *Candida albicans* contributes to differentiation of Kupffer cells or monocytes into anti-inflammatory M2 type macrophages (43); consistently, SA-Pa from *E. gracilis* in this study promoted M2 macrophage activation upon ALI (**Figure 4**). Also, LPS has been reported to induce ATP release from monocytes and contribute to T-cell suppression by generating elevated systemic ATP level that interferes with T-cell metabolism and functions (44). Here, SA-Pa treatment was also found to mitigate T-cell suppression induced by LPS/D-Gal to restore host immune function (**Figure 4**). One possibility is that SA-Pa may disturb LPS-induced ATP release from monocytes. Furthermore, SA-Pa also attenuates the overwhelming activation of infiltration of neutrophils, which also induces liver damage (**Figure 4**). Therefore, SA-Pa restored host immune function upon acute injury by regulating multiple immune cells and improving liver microenvironment.

The hyper-inflammatory state induced by LPS/D-Gal mediated the production of inflammatory cytokines (34). In turn, these inflammatory cytokines stimulate hepatocytes and Kupffer cells to produce IL-6, which induces acute-phase protein production *via* STAT3 activation (45). Consistently, LPS/D-Gal injury induced the STAT3 activation and its downstream gene transcription; meanwhile, SA-Pa reduced the transcriptional levels of STAT3 target genes (**Figure 5**).

## Conclusion

As a safe and long-term used nutritional supplement, Pa from *E. gracilis* is optimized to SA-Pa with a smaller molecular weight. Pa and SA-Pa both have some effects against LPS/D-gal-induced ALI, with SA-Pa showing greater beneficial effects. Mechanistically, SA-Pa treatment promoted M2 macrophage activation that enhanced the anti-inflammatory function in the liver, downregulated STAT3 target genes, as well as alleviated apoptosis and necroptosis caused by the ALI. Attention could be paid to the potential application of SA-Pa as dietary supplementation against liver injury, while its effects on chronic liver injury await further investigation.

## Data Availability Statement

The original contributions presented in the study are included in the article/**Supplementary Material**. Further inquiries can be directed to the corresponding authors.

## Ethics Statement

The animal study was reviewed and approved by the Wuhan University Animal Ethics Committee with the approval number (WDSKY0201705-2).

## Author Contributions

LZ and JW conceived/designed the experiments and wrote themanuscript. YX, JL, HQ, QW, ZC, and CL performed the experiments and data analysis.

## Funding

The authors acknowledge support from the National Key R&D Program of China (2020YFA0908703 and 2018YFA0902500) and the Natural Science Foundation of Guangdong Province, China (2021A1515011155).

## Conflict of Interest

The authors declare that the research was conducted in the absence of any commercial or financial relationships that could be construed as a potential conflict of interest.

## Publisher’s Note

All claims expressed in this article are solely those of the authors and do not necessarily represent those of their affiliated organizations, or those of the publisher, the editors and the reviewers. Any product that may be evaluated in this article, or claim that may be made by its manufacturer, is not guaranteed or endorsed by the publisher.

## Abbreviations

ALI, acute liver injury; LPS, lipopolysaccharide; D-Gal, D-galactosamine; Pa, paramylon; SA-Pa, sonicated and alkalized paramylon; ALT, alanine transaminase; AST, aspartate transaminase; JAK, Janus kinase; STAT, signal transduction and activator of transcription; TUNEL, terminal deoxynucleotidyl transferase-mediated dUTP nick-end labeling; IL-6, interleukin-6; IL-8, interleukin-8; IL-10, interleukin-10; IL-1β, interleukin-1β; TNFα, tumor necrosis factor α; SOCS3, suppressor of cytokine signaling 3.
