# Supplementary material for: Paramylon from Euglena gracilis Prevents Lipopolysaccharide-Induced Acute Liver Injury
Source: Front Immunol. 2022 Jan 11;12:797096. doi: 10.3389/fimmu.2021.797096 (PMC8812190; doi:10.3389/fimmu.2021.797096)
Supplement: Supplementary file 1 [file Image_1.pdf]

Supplementary Figures:

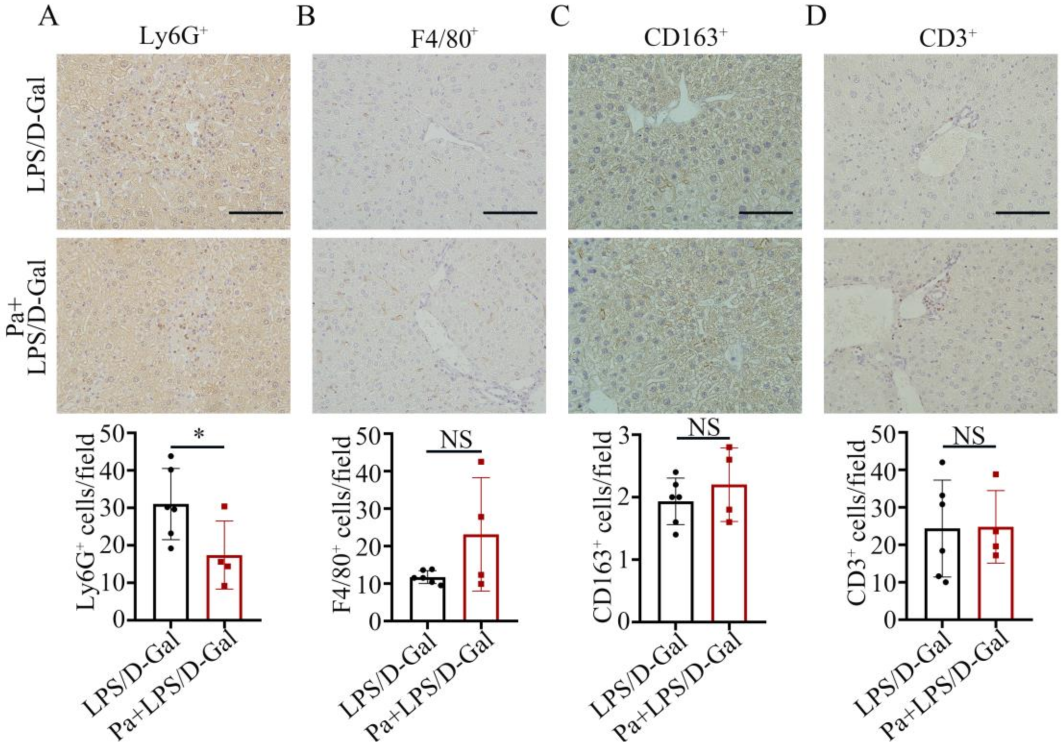

Supplementary Figure 1. The effects of Pa on immune cell infiltration in the liver induced by LPS/D-Gal injury. (A-D) Representative pictures for Ly6G$^+$ (A), F4/80$^+$ (B), CD163$^+$ (C) and CD3$^+$ (D) staining in the liver section (top) with quantitative analysis (bottom) (LPS/D-Gal group, n = 6; Pa + LPS/D-Gal group, n = 4; *$p < 0.05$; NS: no significance; scale bar = 100 μm).

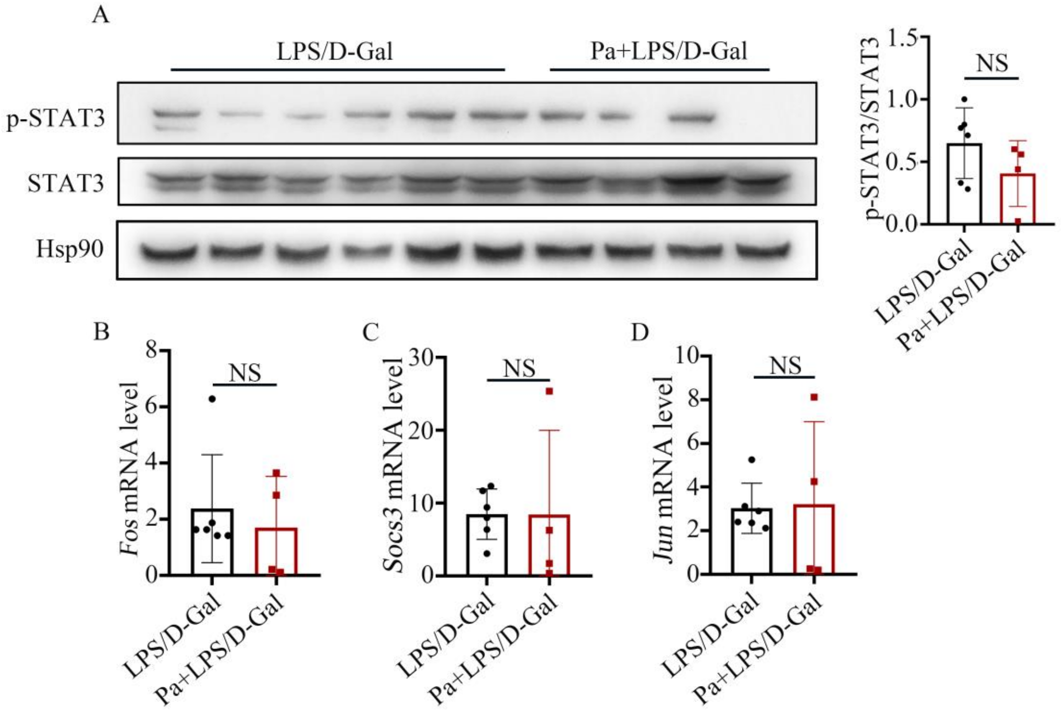

Supplementary Figure 2. Pa had no effect on transcription activity of STAT3 under LPS/D-Gal injury. (A) Representative western blots (left) with quantitative results (right) for p-STAT3/STAT3 level in the liver of indicated groups. (B-D) qPCR results of the indicated genes in the liver of different groups (LPS/D-Gal group, n = 6; Pa + LPS/D-Gal group, n = 4; NS: no significance).

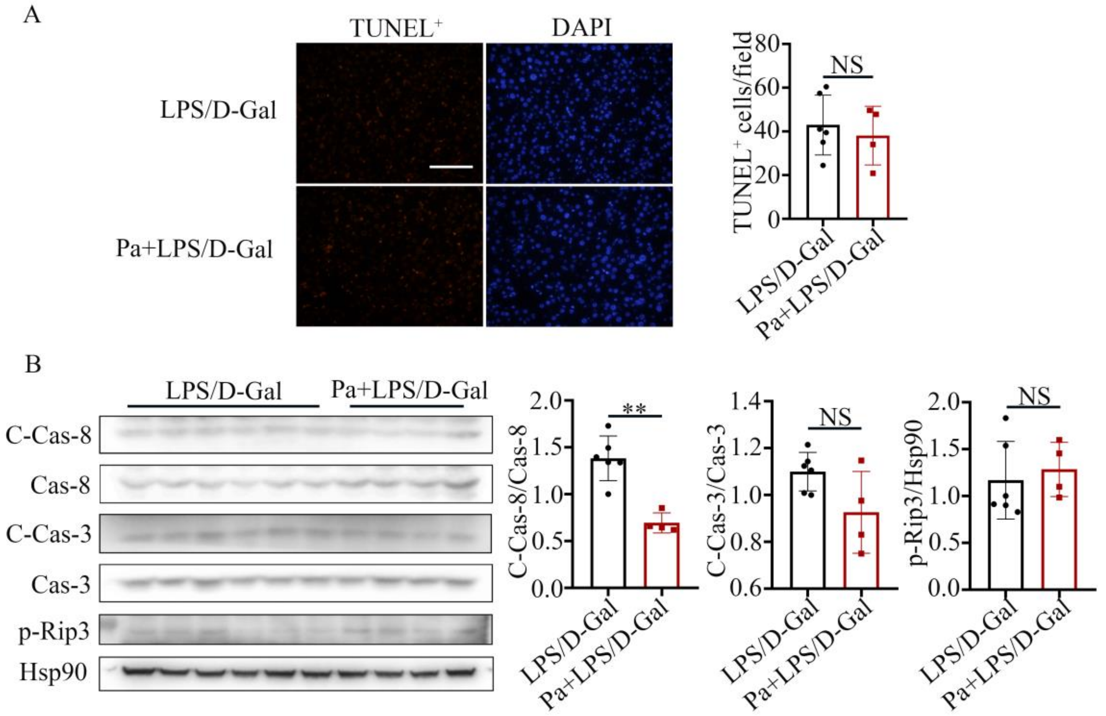

Supplementary Figure 3. The effects of Pa on hepatic cell death under LPS/D-Gal injury. (A) Representative pictures for $TUNEL^+$ staining in the liver section (left) with quantitative results (right). (B) Representative western blots (left) and quantitative results (right) for C-Casp-8/Cas-8 (cleaved-Caspase-8/Caspase-8), C-Cas-3/Cas-3, and p-Rip3 levels. (LPS/D-Gal group, n = 6; Pa group, n = 4; ***p* < 0.01; NS: no significance; scale bar = 100 μm).
